# Competitive PCR with dual fluorescent primers enhances the specificity and reproducibility of genotyping animals generated from genome editing

**DOI:** 10.1186/s13578-023-01042-2

**Published:** 2023-05-11

**Authors:** Liezhen Fu, Emily Ma, Morihiro Okada, Yuki Shibata, Yun-Bo Shi

**Affiliations:** Section On Molecular Morphogenesis, National Institute of Child Health and Human Development, National Institutes of Health, Bethesda, MD 20892 USA

**Keywords:** Genome editing, Genotyping, Fluorescent PCR, *Xenopus tropicalis*

## Abstract

Targeted genome editing is a powerful tool for studying gene function in almost every aspect of biological and pathological processes. The most widely used genome editing approach is to introduce engineered endonucleases or CRISPR/Cas system into cells or fertilized eggs to generate double-strand DNA breaks within the targeted region, leading to DNA repair through homologous recombination or non-homologous end joining (NHEJ). DNA repair through NHEJ mechanism is an error-prone process that often results in point mutations or stretches of indels (insertions and deletions) within the targeted region. Such mutations in embryos are germline transmissible, thus providing an easy means to generate organisms with gene mutations. However, point mutations and short indels present difficulty for genotyping, often requiring labor intensive sequencing to obtain reliable results. Here, we developed a single-tube competitive PCR assay with dual fluorescent primers that allowed simple and reliable genotyping. While we used *Xenopus tropicalis* as a model organism, the approach should be applicable to genotyping of any organisms.

**Supplementary Information:**

The online version contains supplementary material available at 10.1186/s13578-023-01042-2.

**Dear Editor**,

We would like to report a simple and faithful dual color assay for genotyping organisms generated from genome editing. Targeted genome editing is a powerful tool for studying the gene function and underlying molecular mechanisms of almost every aspect of biological and pathological processes. The most popular methods for targeted genome editing are engineered endonucleases, including Zinc Finger Nuclease (ZFN), Transcription Activator-Like Effector Nucleases (TALENs), and Clustered Regularly Interspaced Short Palindromic Repeats/Cas (CRISPR/Cas) system, the prevailing genome editing tool originating from the adaptive immunity system of prokaryotes [1]. The common mechanism of these genome editing systems is to guide a nuclease to a target region to generate double strand DNA breaks within the targeted region, which in turn activates cellular repair system to repair DNA damages through homologous recombination or non-homologous end joining (NHEJ). When NHEJ occurs, point mutations or nucleotide insertions or deletions (indels) are introduced to the targeted region due to its error-prone repair mechanism. Newly introduced in-frame stop codons that prematurely terminate endogenous protein translation and out-of-frame mutations that disrupt endogenous protein synthesis are most useful for studying gene function. Numerous strategies have been developed to estimate the mutation spectrum and efficiency of genome editing by genotyping the resulting organisms, such as the Surveyor assay [2], a two-color fusion protein assay [3], and DNA cloning and sequencing analysis. These assays are typically time consuming and/or inaccurate, especially for genotyping individual organisms with point mutations or short indel mutations generated from genome-editing. Here, we report a simple and accurate dual fluorescence PCR approach for genotyping organisms with point mutations or short indel mutations by using the anuran *Xenopus tropicalis* as an example.

We have been studying frog metamorphosis as a model for mammalian postembryonic development and organ regeneration, a period when plasma thyroid hormone (T3) level peaks and adult organ development and maturation occur [4–6]. Anuran metamorphosis involves drastic changes in essentially all larval tissues in a process that is controlled by T3. This process offers a unique opportunity to identify and functionally characterize genes that are regulated by T3 and, thus, likely play critical roles in the development of adult organs, including organ-specific stem cells. To perform functional analysis of such T3-regulated genes, we applied genome-editing approaches to knock out selected target genes and found that the resulting mutant tadpoles frequently contained short indels (Additional file 1: Fig. S1A–D), which make it difficult to use a PCR approach that relies on the size difference of PCR fragments from the wild type and mutant alleles or uses allele-specific primers for allele-specific amplifications. For example, the gene-edited mutants of the two T3-target genes histidine ammonia-lyase 2 (HAL2) and methyl-CpG binding domain protein 3 (MBD3), contained short indels (Additional file 1: Fig. S1). For each gene, we designed primer pairs with one primer specific for the wild type or mutant alleles and one common primer. Each primer pair was used for PCR amplification on both wild type and homozygous mutant genomic DNAs. The results showed that for HAL2 and MBD3 genes, there were non-specific amplification by the wild type primer pair on homozygous mutant genomic DNA and/or mutant primer set on wild type DNA (Additional file 1: Fig. S2), limiting the ability to accurately determine the genotypes. We tried multiple strategies, such as moving the positions of primers, reducing the primer length and/or raising the annealing temperature during PCR, without reliably improving the specificity sufficiently for accurate genotyping (data not shown).

To overcome this problem, we hypothesized that a perfectly matched primer (e.g., wild type allele-specific primer on wild type DNA template) would compete more effectively against a primer with mismatches (e.g., wild type allele-specific primer on mutant DNA template) when present in the same PCR reaction. Thus, we labelled the mutant-allele specific reverse primer Rm and the wild type allele-specific reverse primer Rwt for HAL2 gene with fluorescent dye IR700 and IR800, respectively. We mixed both primers together in a single tube PCR assay in the presence of the common forward primer F428 (Additional file 1: Fig. S1) on genomic DNA from animals of different HAL2 genotypes. The PCR products were resolved on a PAGE gel, and the gel was scanned for both green fluorescence (IR700) and red fluorescence (IR800). As expected, the PCR on heterozygous template DNA produced an amplified band in both the red fluorescent channel (products amplified with IR800-labeled Rwt) and the green fluorescence channel (products amplified with IR700-labeled Rm). When the images from both channels were merged, a yellow band was produced (Fig. 1A). In contrast, the same single tube PCR with dual fluorescent primers produced a band in the red fluorescent channel on wild type template or in the green fluorescence channel on homozygous mutant templates. These bands remained red or green in the merged images, allowing the three genotypes to be easily distinguishable based on merged images (Fig. 1A). Similarly, when the IR700-labelled MBD3 mutant allele-specific forward primer, Fm, and IR800-labelled MBD3 wild type allele-specific forward primer, Fwt, were mixed together to amplify genomic DNA from animals of different MBD3 genotypes with a common reverse primer in a similar single tube PCR assay, we again found that the three genotypes could be reliably distinguished in the merged images (Fig. 1B). In addition, there were no detectible non-specific amplification by the mutant primer on wild type DNA or vice versa for either gene (Fig. 1).

**Fig. 1:**
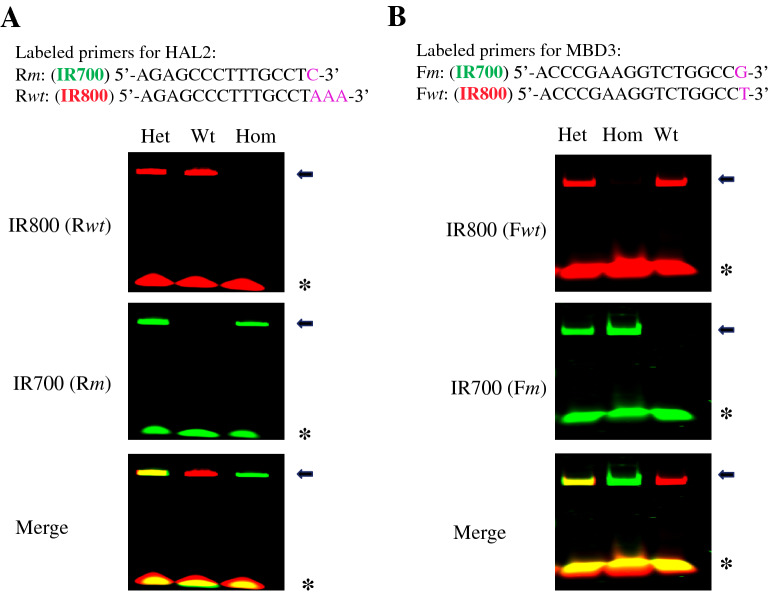
Co-PCR with two genotype-specific fluorescent primers faithfully distinguishes the three genotypes of a genome-editing target gene. Primers labelled with fluorescent IR700 and IR800 dyes that were specific for the wild type and mutant alleles of indicated target gene, respectively, were mixed together with a third primer common to both wild type and mutant alleles in single tube for competitive PCR to genotype animals with mutation in HAL2 **A** and MBD3 **B**. The PCR products were denatured and resolved on 15% Urea-PAGE gels. Fluorescent bands were digitally visualized on a LI-COR Odyssey Clx Scanner with the IR700 signal recorded as green and IR800 signal as red. The fluorescent densities were adjusted in each individual channel to the condition where the green and red fluorescent densities on PCR products of heterozygous mutant targets (Het) were about equal, which generated a yellow band in the merged field. The wild type (Wt) and homozygous mutant (Hom) bands were red and green, respectively. Note that genotype-specific primers could be either reverse primers (Rm and Rwt in panel **A**) or forward primers (Fm and Fwt in panel **B**), and should target the same region with only a short stretch of sequences different at the 3’-end (in purple letters). The arrows point to the PCR products and the star * indicates the unincorporated primers

To test whether primer competition enhanced the PCR specificity in the single tube PCR assay with dual fluorescent primers, we first carried out parallel PCR reactions to genotype HAL2 and MBD3 animals using a single pair of non-fluorescent primers per reaction on serially diluted target DNA templates. We observed significant non-specific amplifications (Additional file 1: Fig. S2), making it difficult to draw conclusions on their genotypes. Next, we carried out single tube PCR with dual fluorescent primers on serially diluted target DNA templates and found that non-specific amplification was eliminated or drastically reduced, allowing for easy identification of the genotypes (Additional file 1: Fig. S3). To further confirm that primer competition in single tube PCR increased PCR specificity, we performed parallel PCR reactions on DNA from animals of all three MBD3 genotypes with the common reverse primer and the genotype-specific fluorescent forward primers both individually and together. The results showed that the wild type allele-specific primer amplified the target region from all three genotypes (Fig. 2, lane 2–4), indicating significant non-specific amplification on the homozygous mutant DNA when used alone with the common reverse primer (Fig. 2, lane 4). Similarly, the mutant allele-specific fluorescent primer non-specifically amplified the wild type target when used alone with the common reverse primer, though at relatively low efficiency (Fig. 2, lane 5). However, in single tube PCR with dual fluorescent primers, the non-specific bands were no longer detectable (Fig. 2, lane 8–10). These results confirmed that the competition of the two allele-specific fluorescent primers in the single tube PCR assay effectively reduced their respective non-specific priming, thus improving the assay specificity for accurate genotyping.

**Fig. 2:**
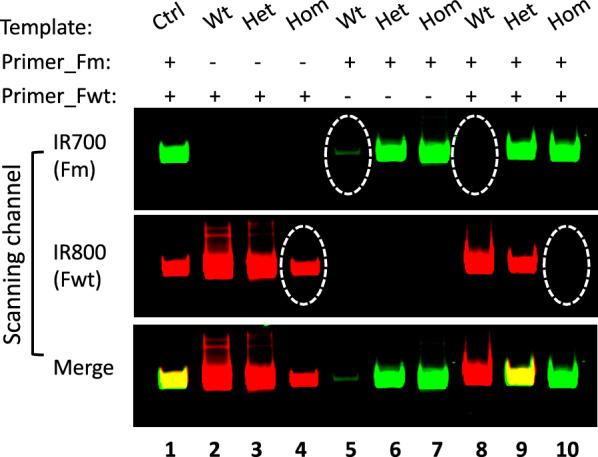
Genotype-specific PCR primers compete for targets to increase amplification specificity. Fluorescently labeled genotype-specific forward primers for MBD3 were used to pair with a common reverse primer individually or in combination in PCR reactions to amplify the gene-editing target region of MBD3 in genomic DNA isolated from wild type (Wt), heterozygous mutant (Het), and homozygous mutant (Hom) animals, respectively. The PCR products were resolved on a gel and scanned to visualize the fluorescent signals as in Fig. 1. Note that the wild type-specific forward primer (Fwt) non-specifically amplified the target region in the homozygous mutant template to produce a strong band when paired alone with the common reverse primer (circled in lane 4). However, the band was absent when both wild type-specific forward primer and mutant-specific forward primer were present together in a single tube dual fluorescent PCR reaction (circled in lane 10), likely due to more effective competition of the mutant-specific forward primer to bind to the mutant templates for PCR amplification. Similarly, the weak non-specific amplification of mutant-specific primer on the wild type template (circled in lane 5) when wild type-specific forward primer was absent was also inhibited in the dual fluorescent PCR reaction when the wild type-specific forward primer was also present (circled in lane 8). Ctrl: control PCR of DNA template from heterozygous animals as reference to adjust green and red signals for visualization

Genome editing is a powerful tool used to introduce mutations in targeted genes to study their function and underlying molecular mechanisms. Faithful and simple genotyping is key for the widespread use of such technology and to ensure correct interpretation of the experimental findings. PCR-based detection is an appealing approach as it works reliably on mutations with large indels that lead to significant size differences in the PCR products from wild type and mutant alleles. Additionally, it has the ability to amplify specific alleles with allele-specific primers. However, it is very difficult to design allele-specific primers with high specificity for organisms with small indels or point mutations, which are common mutant types from genome editing. In such cases, PCR often has non-specific amplifications, making this approach unreliable for genotyping. Targeting a region harboring a restriction enzyme recognition site that can be disrupted via genome editing expands the application of PCR-based detection on small indels but requires additional steps to purify and digest the PCR products prior to resolving them through electrophoresis. In recent years, a number of strategies have been developed to genotype organisms with such small indels with limited practical use due to complex designs and/or lengthy assays [7–10]. Our strategy here uses a single tube PCR assay containing two allele-specific primers labelled with different fluorescent dyes to pair with a common primer for wild type and mutant alleles (note that while we used IR700 and IR800 fluorescent dyes to label the primers to visualize PCR products through a LI-COR Odyssey Clx Scanner, any combination of other fluorescent dyes can be used to visualize the bands with a compatible scanner). The two allele-specific primers differ slightly at the 3’-end and competitively prime the same region of the templates for PCR amplification. The PCR products from wild type and a mutant allele with a small indel or point mutation(s) have the same or very similar sizes and thus will co-migrate on a denaturing PAGE gel (Fig. 1) or an alkaline agarose gel (Additional file 1: Fig. S4), allowing easy genotype judgement based on merged image color. Due to primer competition in the single tube PCR assay, the specificity was significantly improved to avoid error in genotype determination. Thus, the single-tube PCR assay using dual allele-specific fluorescent primers provides a simple but reliable strategy to genotype germline-transmissible mutations for different organisms and even cell lines. If no available fluorescent recording system is available, the single-tube competitive PCR assay can still enhance the specificity of genotyping that uses non-labelled primers and takes advantage of PAGE gel’s high resolution in differentiating the allele-specific PCR products with small size differences.

## Materials and methods

These are shown in the supplemental information.

## Supplementary Information

**Additional file 1.** Comparative analysis of regular PCR and competitive single-tube dual fluorescent PCR for genotyping small deletion mutant X. tropicalis animals generated via CRISPR/Cas9-mediated genome editing. Regular PCR with a single genotype-specific primer set in a PCR reaction often leads to non-specific amplification and inconclusive or false genotyping results. On the other hand, competitive single-tube PCR with a mixture of two genotype-specific fluorescent primers and a common primer inhibits non-specific amplification to allow accurate genotyping.

## Data Availability

Not applicable.

## Abbreviations

PCR: Polymerase chain reaction
*X. tropicalis*: *Xenopus tropicalis*
ZFNases: Zinc finger nuclease
TALENs: Transcription activator-like effector nucleases
CRISPR/Cas: Clustered regularly interspaced short palindromic repeats/Cas
sgRNA: Short guide RNA
NHEJ: Non-homologous end joining
HAL2: Histidine ammonia-lyase 2
MBD3: Methyl-CpG binding domain protein 3
PAGE: Polyacrylamide gel electrophoresis
