# Supplementary material for: Competitive PCR with dual fluorescent primers enhances the specificity and reproducibility of genotyping animals generated from genome editing
Source: Cell Biosci. 2023 May 11;13:83. doi: 10.1186/s13578-023-01042-2 (PMC10173569; doi:10.1186/s13578-023-01042-2)
Supplement: Supplementary file 1 — Additional file 1. Comparative analysis of regular PCR and competitive single-tube dual fluorescent PCR for genotyping small deletion mutant X. tropicalis animals generated via CRISPR/Cas9-mediated genome editing. Regular PCR with a single genotype-specific primer set in a PCR reaction often leads to non-specific amplification and inconclusive or false genotyping results. On the other hand, competitive single-tube PCR with a mixture of two genotype-specific fluorescent primers and a common primer inhibits non-specific amplification to allow accurate genotyping. [file 13578_2023_1042_MOESM1_ESM.pdf]

ADDITIONAL MATERIALS AND METHODS

Experimental animals

Wild type adults *X. tropicalis* were purchased from NASCO. Embryos and tadpoles were generated as described [1]. All animal care and treatments were performed as approved by the Animal Use and Care Committee of Eunice Kennedy Shriver National Institute of Child Health and Human Development of the National Institutes of Health.

Gene editing and germline breeding

Short guide RNA (sgRNA) for guiding CRISPR/Cas9 to genome targets were designed as previously described [1] to target the exon 4 of *X. tropicalis* HAL2 [2] and exon 1 of *X. tropicalis* MBD3 [3], respectively. Wild type embryos were injected with CRISPR/Cas9 mRNA and sgRNA mix at one-cell stage, screened for the tadpoles with high rates of out-of-frame mutations as described [4], and reared to adulthood (F0 generation frogs). Sexually mature F0 frogs were used one at a time to mate with a wild type frog to produce F1 generation tadpoles to obtain heterozygous mutant tadpoles harboring out of frame mutations within the targeted exon as described [4]. The mutant animals were further confirmed through PCR cloning the target region and sequencing and raised to adulthood (F1 heterozygous frogs). A pair of F1 heterozygous frogs were mated to produce F2 generation tadpoles.

DNA extraction and genotyping

F2 generation tadpoles after onset of feeding (stage 45) were used to tail tip clipping (3 to 5 mm) to obtain genomic DNA for genotyping as previously described [5]. The genomic DNA was subjected to first round PCR with primer pairs encompassing the target regions, common to both wild type and mutant alleles (Supplemental Fig. 1 and Supplemental Table 1) and the products were diluted at 1:1000 to serve as templates for regular PCR genotyping by using genotype-specific primers or diluted into 100 pg/μL followed by serial dilution at 1:5 to serve as templates to test PCR specificity at different concentrations of templates. For fluorescent PCR, the IR700- and IR800-labeled primers (custom-designed and purchased from Intergrated DNA Technologies, Coralville, IA) were mixed with their unlabeled respective primers of same sequences at 1:50 for use in PCR reactions, with the final concentration of each primer at 0.5 μM. PCR reactions were done with conventional Taq DNA polymerase (Thermo Fischer Scientific, Waltham, MA).

Gel electrophoresis and DNA detection

Regular PCR products from unlabeled primers were resolved on a 2% agarose gel, stained with ethidium bromide, and photographed with a digital camera attached to AlphaImager HP (ProteinSimple, Minneapolis, MN). Fluorescent PCR products were denatured by mixing with an equal volume of 2 × Urea Loading Buffer (20mM Tris-HCl, pH8.0, 1 mM EDTA, 8M urea, 0.05% Orange G), resolved on polyacrylamide gels (15% TBE-Urea PAGE Gel, Thermo Fischer Scientific, Waltham, MA), and digitalized by scanning the gel on LI-COR Odyssey Clx Scanner with the IR700 recorded as green and IR800 as red signals.

References

1. Shibata Y, Bao L, Fu L, Shi B, Shi YB: **Functional Studies of Transcriptional Cofactors via Microinjection-Mediated Gene Editing in Xenopus**. *Methods Mol Biol* 2019, **1874**:507-524.
2. Luu N, Fu L, Fujimoto K, Shi YB: **Direct Regulation of Histidine Ammonia-Lyase 2 Gene by Thyroid Hormone in the Developing Adult Intestinal Stem Cells**. *Endocrinology* 2017, **158**(4):1022-1033.
3. Fu L, Li C, Na W, Shi YB: **Thyroid hormone activates Xenopus MBD3 gene via an intronic TRE in vivo**. *Front Biosci (Landmark Ed)* 2020, **25**:437-451.
4. Fu L, Wen L, Luu N, Shi YB: **A simple and efficient method to visualize and quantify the efficiency of chromosomal mutations from genome editing**. *Sci Rep* 2016, **6**:35488.
5. Shibata Y, Wen L, Okada M, Shi YB: **Organ-Specific Requirements for Thyroid Hormone Receptor Ensure Temporal Coordination of Tissue-Specific Transformations and Completion of Xenopus Metamorphosis**. *Thyroid* 2020, **30**(2):300-313.

Additional Table S1

Primer	Sequences	Target gene	Genotype-specificity
F428	5’-CGCTATTTACATTGGAGTTCTGGTGAATAAGGCA-3’	HAL2	Both wild type and mutant
R429	5’-TGCTGCACTTTGTTTTCAGCTTGTGGAGT-3’	HAL2	Both wild type and mutant
R*wt*	5’-AGAGCCCTTTGCCTAAA-3’	HAL2	Wild type
R*m*	5’-AGAGCCCTTTGCCTC-3’	HAL2	Mutant
F	5’-CCGGCAGCGTGGTCCTGCTATC-3’	MBD3	Both wild type and mutant
R	5’-CTCTTTATTCCTCCAGCTGCACC-3’	MBD3	Both wild type and mutant
R*wt*	5’-ACCCGAAGGTCTGGCCT-3’	MBD3	Wild type
R*m*	5’-ACCCGAAGGTCTGGCCG-3’	MBD3	Mutant

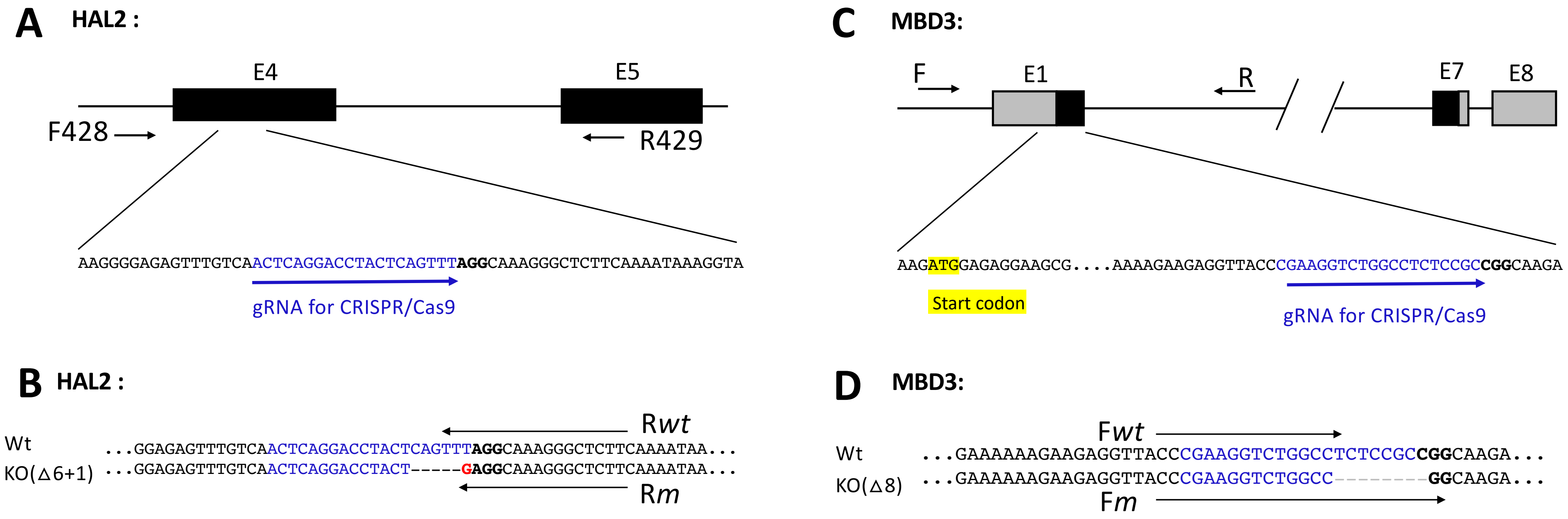

Additional Fig. S1. Small deletion mutants generated in *X. tropicalis* by CRISPR/Cas9-mediated genome editing.

A and C. Schematic diagrams for CRISPR/Cas9-mediating gene editing of *X. tropicalis* HAL2 (A) and MBD3 (C) genes. CRISPR/Cas9 guide RNAs for HAL2 (A) and MBD3 (C) were designed to target the coding sequence in exon 4 of HAL2 gene and the first exon of MBD3, respectively, and synthesized through *in vitro* transcription. Each gRNA was mixed with CRISPR/Cas9 mRNA and injected into fertilized *X. tropicalis* eggs at one-cell stage. The resulting embryos were raised to adulthood to produce germline mutant animals. Solid boxes: coding sequences in exons; grey boxes: noncoding sequences in exons; blue letters: gRNA target sequences with the protospacer adjacent motif (PAM) sequences in bold; and blue arrows; the direction of the gRNA orientation. F428 and R429 for HAL2 (A) and F and R for MBD3 (C) were PCR primers for amplifying the respective target regions.

B and **D**. Specific primers for wild type and mutant alleles generated by CRISPR/Cas9-mediated gene editing of HAL2 (B) and MBD3 (D). Dots (.):DNA sequences not shown for simplicity; dashes (-): deletions in the mutant; red letter: point mutation in the mutant; arrows: allele-specific primers for genotyping. Rwt and Rm are reverse primers specific for wild type and mutant HAL2, respectively; Fwt and Fm are forward primers specific for wild type and mutant MBD3, respectively.

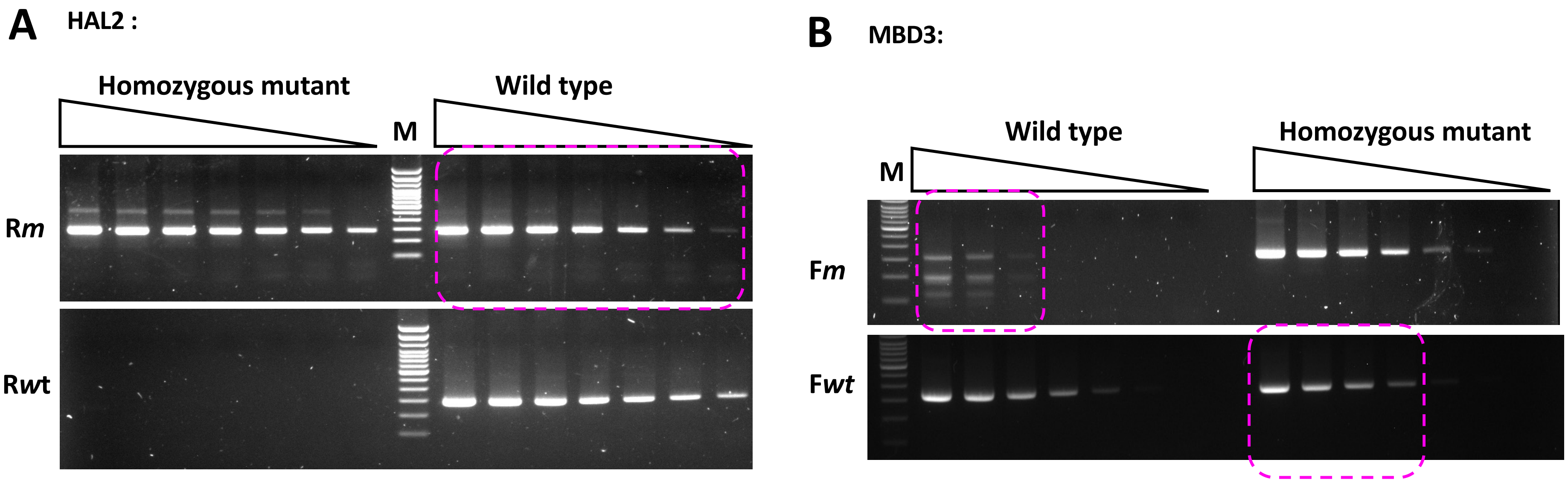

Additional Fig. S2. PCR with a single genotype-specific primer set often leads to non-specific amplification and inconclusive or false genotyping results.

A. Homozygous mutant or wild type HAL2 target region was amplified with the primer pair with F428 and R429 (Supplemental Fig. 1) from the DNA of a homozygous mutant or wild type animal, respectively. The amplified DNA was diluted into 100 pg/ul and further diluted serially at 1:5. The resulting DNA solutions were subjected to parallel PCR amplifications with the common forward primer F428 and allele-specific reverse primer Rm or Rwt. The PCR products were separated on an agarose gel, stained with ethidium bromide, and photographed. Note that the mutant-specific primer set was able to amplify the wild type DNA non-specifically (pink box, top right), although less efficiently (compare the last two lanes of the top and bottom panels).

B. Homozygous mutant or wild type MBD3 target region was amplified with the primer pair with F and R (Supplemental Fig. 1) from the DNA of a homozygous mutant or wild type animal, respectively. The amplified DNA was diluted into 100 pg/ul and further diluted serially at 1:5. The resulting DNA solutions were subjected to parallel PCR amplifications with allele-specific forward primer Fm or Fwt and the common forward primer R. Note that the mutant-specific primer set was able to amplify the wild type DNA non-specifically (purple box, top left) while the wild type-specific primer set was also able to amplify the mutant DNA non-specifically (purple box, bottom right).

The non-specific PCR products (in purple boxes) made it impossible to determine the genotype correctly. M: DNA marker.

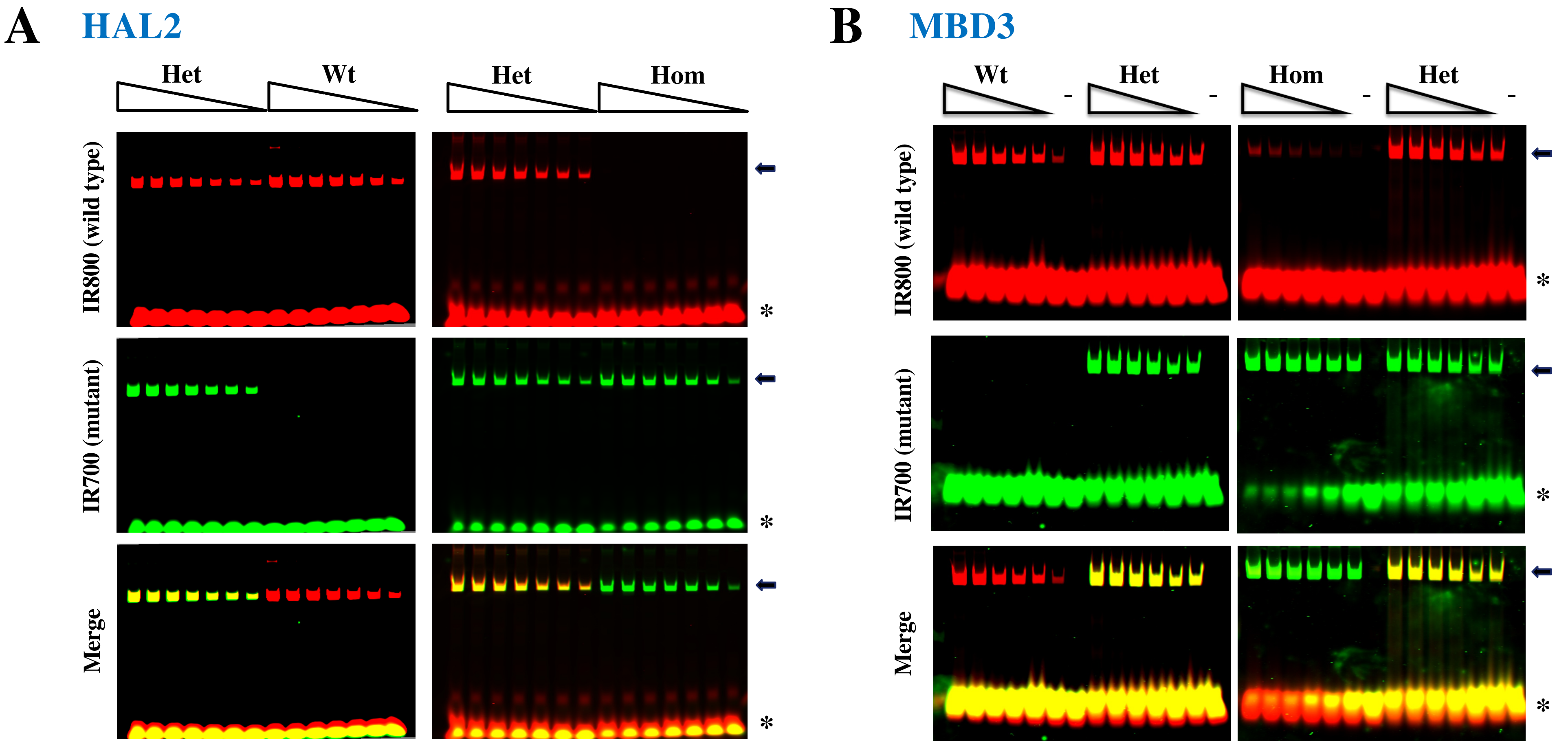

Additional Fig. S3. Competitive single-tube PCR with a mixture of two genotype-specific fluorescent primers and a common primer inhibits non-specific amplification to faithfully identify genotypes at a wide range of template concentrations.

Serially diluted templates of wild type (Wt), heterozygous (Het) and homozygous (Hom) mutant HAL2 (A) and MBD3 (B) as described in Supplemental Fig. 2 were subjected to single-tube PCR by using two genotype-specific fluorescent primers together with a primer common to both wild type and mutant alleles. The PCR products were resolved on a gel and scanned to visualize the fluorescent signals as described in Fig 1. Note the absence or drastically reduced non-specific amplification allowed faithful genotyping based on merged photos (red for wild type, green for homozygous, and yellow for heterozygous) at all template concentrations. The arrows point to the PCR products and the star * indicates unincorporated primers.

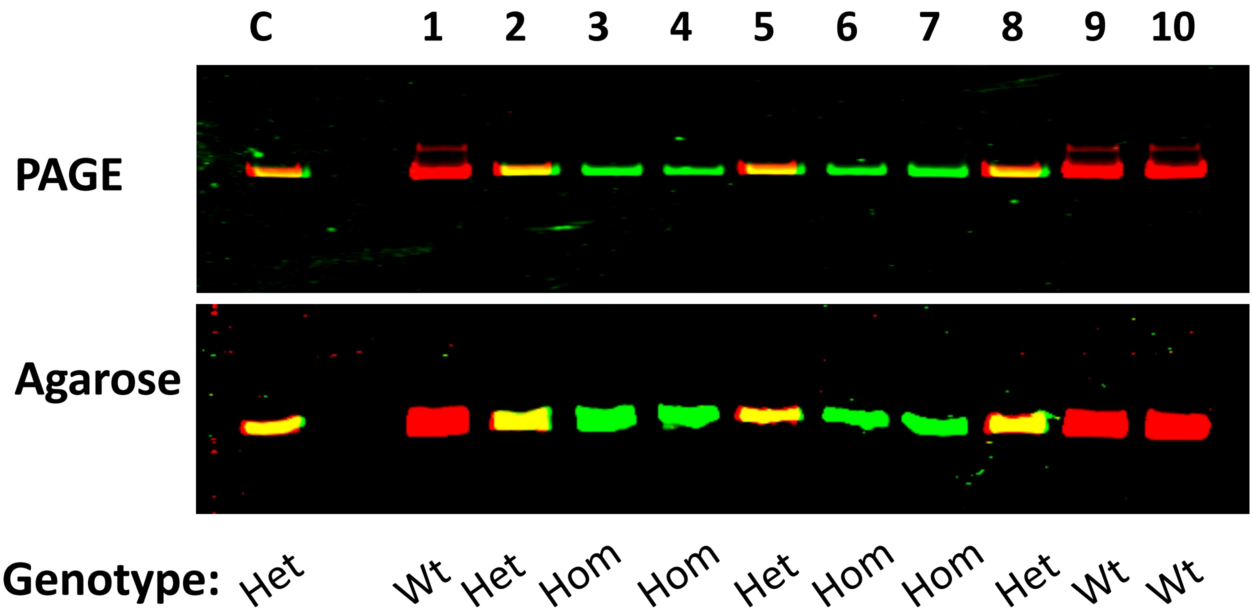

Additional Fig. S4. Both PAGE and agarose gels can be used for dual fluorescent PCR genotyping.

Competitive single-tube PCR with a mixture of two genotype-specific fluorescent primers and a common primer for MBD3 was done as described in Fig. 1B. The PCR products were denatured and separated by electrophoresis on a 15% urea PAGE gel (PAGE, Thermo Fischer Scientific) or a 3% alkaline agarose gel (Agarose). The alkaline agarose gel was prepared by melting agarose powder in a buffer containing 50 mM NaCl and 1 mM EDTA, pouring the resulting gel solution into a gel tray to solidify, and then submerging the gel into an alkaline solution of 50 mM NaOH and 1 mM EDTA for at least 30 min. After electrophoresis, the fluorescent bands were digitally visualized on a LI-COR Odyssey Clx Scanner. The colors of the whole gel were adjusted as described in Fig. 1B such that the green and red fluorescent signals of the PCR products on a known heterozygous (Het) DNA sample (the control, C) were about equal, thus yielding a yellow band. Note that the analyses on the PAGE and alkaline agarose gels yielded identical result for every sample (1 to 10). Wt: wild type; Hom: homozygous.
